# Comparative genetic diversity in a sample of pony breeds from the U.K. and North America: a case study in the conservation of global genetic resources

**DOI:** 10.1002/ece3.1562

**Published:** 2015-07-31

**Authors:** Clare L Winton, Yves Plante, Pamela Hind, Robert McMahon, Matthew J Hegarty, Neil R McEwan, Mina C G Davies-Morel, Charly M Morgan, Wayne Powell, Deborah M Nash

**Affiliations:** 1 IBERS, Aberystwyth UniversityAberystwyth, Ceredigion, SY23 3DA, U.K; 2 Agriculture and Agri-Food CanadaSaskatoon, Saskatchewan, S7N 5A8, Canada; 3 CGIARF-34394, Montpellier Cedex 5, France

**Keywords:** Conservation, founder effect, horse, microsatellite, mtDNA, phylogenetics

## Abstract

Most species exist as subdivided ex situ daughter population(s) derived from a single original group of individuals. Such subdivision occurs for many reasons both natural and manmade. Traditional British and Irish pony breeds were introduced to North America (U.S.A. and Canada) within the last 150 years, and subsequently equivalent breed societies were established. We have analyzed selected U.K. and North American equivalent pony populations as a case study for understanding the relationship between putative source and derived subpopulations. Diversity was measured using mitochondrial DNA and a panel of microsatellite markers. Genetic signatures differed between the North American subpopulations according to historical management processes. Founder effect and stochastic drift was apparent, particularly pronounced in some breeds, with evidence of admixture of imported mares of different North American breeds. This demonstrates the importance of analysis of subpopulations to facilitate understanding the genetic effects of past management practices and to lead to informed future conservation strategies.

## Introduction

The maintenance of populations to preserve maximal genetic variability and future adaptation potential is a common management challenge of conservation efforts in both domestic and wild species. Every finite population risks losing variability through stochastic sampling particularly when the number of reproducing individuals is restricted, creating founder effects and bottlenecks, for example, during: reintroduction of wild animals to previous habitats, formation of ex situ captive breeding groups, and formation of domestic species (Food and Agriculture Organization, 2007; Shen et al. 2009; Vonholdt et al. 2010). Establishing subpopulations requires sufficient sampling of the founding source's genetic variability to be sustainable and remains representative of the origin (Vonholdt et al. 2010), and small founder numbers and/or limited lineage sampling is associated with increased rates of inbreeding and divergence from the source (Frankam et al. 2002). Maintaining gene flow between, and limiting reproductive variance within, the derived subpopulations is predicted to counteract the negative effects of genetic drift on diversity reduction and adaptive allele loss, particularly for small populations (Miller et al. 2009; Vonholdt et al. 2010). These principles apply universally. However, in many cases, the genetic consequences of current or previous management practices are not well characterized or understood. For domestic animals, divergent selection and gene flow have been occurring on a local and continental scale since prehistoric times, yet formalized breeding organizations or societies have arisen only in the last ∼300 years (Ensminger and Parker 1986; Food and Agriculture Organization, 2007). Subpopulations of recognized breeds have been introduced into countries beyond their native origin, under the influence of commercial organizations, breeders' associations, and hobby enthusiasts (Food and Agriculture Organization, 2007). These introductions have often been documented, but the degree to which daughter populations effectively preserve the genetics of the original stock is often poorly characterized. Because the dynamics of these domesticated groups can be considered as modeling a larger range of conservation issues, genetic investigations to determine how representative subpopulations are of the original stocks in relation to known recorded information can potentially provide insight into future international strategies for conservation.

Mitochondrial DNA and microsatellite markers have been utilized to discover the origins and relationships of many horse breeds (Royo et al. 2005; Solis et al. 2005; Cai et al. 2009; Van de Goor et al. 2011; Prystupa et al. 2012a,b) and to elucidate the domestication process (Vila et al. 2001; Jansen et al. 2002; Cieslak et al. 2010; Lippold et al. 2011; Achilli et al. 2012). Others characterized the concordance between pedigrees and actual genetic relationships of current Thoroughbreds and Arabs (Hill et al. 2002; Glazewska et al. 2007). However, limited data compare populations of the same breed established on different continents. Nevertheless, pony populations derived from U.K. and Irish Native breeds have been examined in diverse locations, for example, maternal origins and mitochondrial diversity of native ponies have been studied in Ireland (McGahern et al. 2006) and North America (Prystupa et al. 2012a,b). Others have incorporated subsets of British and Irish pony breeds as part of a wider dataset for microsatellite (Simple Sequence Repeat; SSR) analysis, although the sample population origins are not always stated or are from exported subpopulations (Luis et al. 2007; Leroy et al. 2009; Van de Goor et al. 2011). There are 11 recognized breeds of pony native to Britain and Ireland plus a distinct feral population living in the Carneddau mountain range of Snowdonia, North Wales (Winton et al. 2013). Prystupa et al. (2012a,b) analyzed samples from a comprehensive range of these mountain and moorland-type pony breeds from North America (U.S.A. and Canada) using mtDNA/SSR markers. As such, an opportunity exists to compare the genetic diversity and structure present within these subpopulations with data from the U.K. source populations. Moreover, a broad study of U.K. and North American native breeds employing comprehensive analyses provides an overarching view of international horse gene flow and divergence, rather than solely focusing on diversity of specific subpopulations of localized breeds.

**Figure 1:**
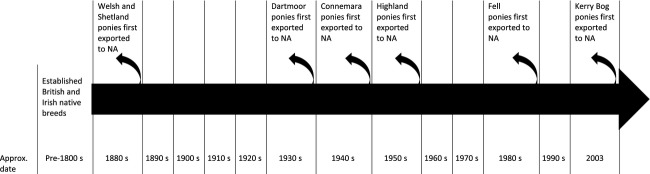
Timeline of breed export to North America (NA). Established British and Irish native breeds include the following: Welsh (sections A and B), Dartmoor (English), Fell (English), Highland (Scottish), Shetland (Scottish), Connemara (Irish), and Kerry Bog (Irish).

Importation of recognized horse breeds into North America started in the 1800s; some breeds have been systematically imported for over 100 years, while others became established more recently (Fig. 1). Welsh pony imports commenced in the 1880s and were first officially recorded in 1909 when six stallions and 27 mares were sent to the U.S.A. and Canada. Imports into the U.S.A. peaked at 427 in 1957, with an annual median of eight animals between 1957 and 1991, although there were also no recorded imports in some years, particularly during wartime (Davies 1993). The importance of this breed in North America is illustrated by the establishment of a formalized breed society in 1907 (Evans 2001). The earliest named shipment of Shetland ponies to the U.S.A. was 1884, and the U.S.A. breed society was established in 1888. High demand for Shetlands existed (1332 ponies imported 1900–1910) and, except for a decline during the World Wars, importation continues to the present day (Russell 1996; Hodson 1997). In contrast, the Kerry Bog Pony has only recently become established in the U.S.A. with an initial breeding herd imported in 2003 (Anon, 2013a).

This study uses mitochondrial DNA sequencing and a panel of microsatellite markers to determine whether North American daughter pony populations maintain genetic diversity while remaining representative of the U.K. source breeds despite different patterns of importation.

## Materials and Methods

### Sample collection and genomic DNA extraction

Studies were undertaken using animals representative of different bloodlines for each of eight British and Irish native pony populations. Populations were referred to using their breed name and those sampled from North America (the U.S.A. and Canada, as described by Prystupa et al. (2012b)) were prefixed with the identifier “U.S.” The Welsh Pony and Cob is subdivided into four distinct sections (A, B, C, and D) defined by the animal's adult height and conformation characteristics (Winton et al. 2013). U.S. Welsh Pony samples were not subclassified, so both U.K. sections A and B were included, as these ponies are more common in North America than the rare Section C. The Section D is a larger animal, often technically classed as a horse. DNA samples were collected as previously described for U.K. and Irish sources by Winton et al. (2013) and North America by Prystupa et al. (2012b). Mitochondrial sequences from 39 Kerry Bog Ponies sampled from Ireland (McGahern et al. 2006) were downloaded from GenBank (http://www.ncbi.nlm.nih.gov/genbank/). Mitochondrial sequence data for the corresponding breeds of North American origin analyzed by Prystupa et al. (2012a) were downloaded from GenBank for analysis. A total of 410 animals were used for the mtDNA analysis and 472 for the microsatellite analysis. See Table 1 for population sample numbers. See Appendix 1 for origin of data and GenBank accession numbers.

**Table 1:** Summary statistics for mtDNA and SSR genotyping for each U.S. and U.K. pony population

Population	*N*	*H*	mtDNA *h ±* SD	*π ±* SD	*N*	AR	SSR *H*_e_	*H*_o_	*F*_IS_
Welsh Section A	47	15	0.925 ± 016	0.0239 ± 0.0013	52	6.14	0.698	0.674	0.021
Welsh Section B	29	12	0.904 ± 0.028	0.0187 ± 0.0030	25	6.05	0.716	0.685	0.028
U.S. Welsh Pony	10	7	0.867 ± 0.107	0.0200 ± 0.0035	48	6.73	0.762	0.748	0.018
Fell	46	14	0.929 ± 0.014	0.0231 ± 0.0013	46	6.73	0.738	0.73	0.003
U.S. Fell	9	2	0.222 ± 0.166	0.0042 ± 0.003	25	5.25	0.683	0.641	0.055
Highland	46	15	0.916 ± 0.017	0.0178 ± 0.0012	40	5.93	0.707	0.722	−0.045
U.S. Highland	11	6	0.836 ± 0.089	0.0233 ± 0.0030	25	4.76	0.657	0.658	−0.006
Connemara	46	18	0.882 ± 0.032	0.0192 ± 0.0019	47	5.90	0.743	0.73	0.010
U.S. Connemara	12	6	0.818 ± 0.096	0.0243 ± 0.0040	37	5.93	0.751	0.778	−0.037
Dartmoor	40	10	0.814 ± 0.037	0.0122 ± 0.0020	39	6.08	0.694	0.685	0.003
U.S. Dartmoor	12	3	0.439 ± 0.158	0.0114 ± 0.0039	25	4.97	0.639	0.609	0.042
Shetland	39	8	0.767 ± 0.054	0.0225 ± 0.0026	35	5.60	0.664	0.657	0.005
U.S. Shetland	14	6	0.835 ± 0.057	0.0220 ± 0.0026	28	5.18	0.643	0.614	0.045
Kerry Bog Pony	39	17	0.934 ± 0.020	0.0210 ± 0.0017	NA	NA	NA	NA	NA
U.S. Kerry Bog Pony	10	5	0.800 ± 0.100	0.0151 ± 0.0028	NA	NA	NA	NA	NA

*N,* sample number; *H*, number of mtDNA haplotypes; *h*, mtDNA haplotype diversity; *π*, mtDNA nucleotide diversity; SD, standard deviation; AR, allelic richness based upon a minimum sample size of 23 individuals; *H*_e_, expected heterozygosity; *H*_o_, observed heterozygosity; and *F*_IS_, inbreeding coefficient.

### Microsatellite genotyping

SSR genotyping of the samples were performed for 17 SSR loci using commercially available kits and protocols as described previously for the U.K., Winton et al. (2013), and the U.S.A. for 38 markers including these 17, Prystupa et al. (2012b). PCR products were sized using a ABI 3730 or 3130xl Genetic Analyzer (Applied Biosystems, Paisley, UK) with the internal 600-Liz size standard. Genotypes were determined using Genemapper® Software v3.0 (Applied Biosystems).

### Microsatellite analysis

To integrate U.S. and U.K. datasets, subsets of samples were exchanged between laboratories for genotyping and allele scoring. Datasets for the overlapping markers used were adjusted accordingly and standardized according to ISAG recommendations. Allele frequencies were examined for significant deviation (*P* < 0.05) from Hardy–Weinberg expectations using the exact test in Arlequin 3.5 (Excoffier et al. 2005; Excoffier and Lischer 2010), with Markov chain length set to 100,000 following 10,000 dememorization steps. Markers displaying highly significant deviations in more than one population and/or containing large amounts of missing data values were removed to limit the influence of allele dropout. Loci with missing data unevenly distributed across the populations (>5% per breed) were also excluded resulting in seven loci being removed from the analysis, including two that had demonstrated null alleles in other studies (Rendo et al. 2012). Individual samples with poor-quality DNA with calls missing from >3 loci were also excluded.

An unbiased Bayesian approach using Markov chain Monte Carlo (MCMC) clustering of samples was conducted via the STRUCTURE v2.2.3 software (Pritchard et al. 2000). Parameters were set as diploid data for each individual and assessed for values of *K* ranging from 1 to 16. Burn-in and MCMC iteration settings were 50,000 and 100,000, respectively. Allele frequencies were treated as correlated. For each value of *K*, six replicate simulations were conducted. The *K* statistic (the second-order rate of change in log probability between successive values of *K*) was calculated using STRUCTURE Harvester v0.6.7 (http://taylor0.biology.ucla.edu/struct_harvest/) as per Evanno et al. (2005). Results from replicate runs at the optimal *K* were combined in CLUMPP (Jakobsson and Rosenberg 2007) and the average Q-table exported to DISTRUCT for graphical presentation (Rosenberg 2004). Population summary statistics, analyses to detect population expansion according to Harpending's raggedness index, pairwise *F*_ST_ comparisons, and analysis of molecular variance (AMOVA) were calculated using Arlequin 3.5 (Excoffier and Lischer 2010). To account for difference in sample size, allelic richness (AR) was calculated for each locus and population based on a minimum sample size of 23 diploid individuals using FSTAT v2.9.3.2 (Goudet 2001).

### mtDNA genotyping

U.K. samples were sequenced for a 606-bp mtDNA fragment of the mitochondrial control region (MTCR as described previously; Winton et al. 2013). Sequence data were trimmed to a 212-bp sequence in order to align the sequence fragment shared with the U.S. samples (Prystupa et al. 2012a). Alignment was performed using BioEdit Sequence Alignment Editor v7.0.9.0 (Hall 1999) before being exported in phylip (.phy) format.

### mtDNA analysis

A median-joining network was created to explore the relationship between haplotypes using NETWORK (http://www.fluxus-engineering.com). In line with other studies, hypervariable nucleotide positions 15,585, 15,597, and 15,650 were removed (downweighted to 0) and nucleotide positions 15,604, 15,659, and 15,737 were downweighted (from 10 to 5). Nucleotide positions refer to that of the complete horse reference sequenced by Xu and Arnason (1994). The 83 reference sequences from Achilli et al. (2012) were downloaded to create a skeleton network of the globally defined horse haplogroups. Additionally, 208 archaeological samples (Cieslak et al. 2010) were obtained from GenBank to identify the distribution of ancestral diversity and its relation to modern populations. Sequence traces were checked for nucleotide call errors and either manually rectified or removed in the case of overall low-quality sequence.

Haplotype number, diversity, and nucleotide diversity were calculated for each population using DnaSP v5 (Librado and Rozas 2009). A haplotype list was generated for each dataset by DnaSP v5 based on the original sequences and used in Arlequin 3.5 (Excoffier and Lischer 2010) to calculate the average pairwise nucleotide differences between each population and to estimate number of migrants exchanged between populations. Mismatch distributions were also analyzed to detect evidence of population expansion.

To infer past population dynamics, such as changes in relative effective population size throughout time indicating evidence of genetic bottlenecks, Nexus files of aligned mtDNA sequences were prepared for each population and used to generate extended Bayesian Skyline plots by Bayesian Monte Carlo simulation of coalescent molecular trees using the phylogenetic inference software package BEAST (Bayesian Evolutionary Analysis Sampling Trees) (Drummond and Rambaut 2007; Heled and Drummond 2010; Drummond et al. 2012). Priors were set to the same values for all populations, with default parameter setting being used with the exception of the prior mutation rate, which was set to a constant of 0.05 mutations per site per million years. These plots provide relative comparisons of molecular evolutionary history dynamics between U.S. and U.K. breeds by estimating the effective number of females throughout a continuous time period based upon the mitochondrial molecular diversity within the current populations. As such, they are not absolute estimates of past population sizes and absolute chronological timings of events. This decision was taken because of the inferred potential for different accuracy in estimation of parameters from the discrepancy in available sample sizes from the two groups. To rule out a consistent bias of sample size in the estimated demographies, a random subset of samples with equivalent numbers of molecules was subsampled from the U.K. populations. These samp-les revealed that, in direct contrast to the U.S. samples, estimates of predomestication population size were similar to those predicted using the larger U.K. population dataset, but with larger confidence intervals.

## Results

### Parameters of population genetic diversity

Summary statistics are presented in Table 1. Analysis of mitochondrial control region (MTCR) sequence from 410 individuals identified 46 haplotypes (DNASP v5) based on 34 variable nucleotide sites (212-bp sequence, DNASP v5). Overall haplotype diversity was high (0.949 ± 0.003) with high nucleotide diversity (0.0232 ± 0.0005). The U.S. Fell population possessed the lowest haplotype/nucleotide diversity of all populations and the lowest number of haplotypes (two). The Kerry Bog group demonstrated the highest haplotypes diversity (0.934) and the U.S. Connemara had the highest overall nucleotide diversity (0.0243), while the Connemara had the highest number of haplotypes (18). With the exception of Shetlands, U.S. populations showed lower mitochondrial diversity than U.K. equivalents, although both U.S. Connemara and U.S. Highland pony populations displayed greater nucleotide diversity values than their U.K. equivalents.

All U.S. populations had lower autosomal diversity than U.K. counterparts according to SSR allelic richness, observed heterozygosity (*H*_O_), and inbreeding coefficients (*F*_IS_), with the exception of the U.S. Welsh Pony and the U.S. Connemara (Table 1). The U.S. Welsh Pony demonstrated the highest values within the entire dataset for allelic richness and high *H*_O_, while the U.S. Connemara exhibited the highest *H*_O_ and low *F*_IS_. For all parameters, the U.S. Dartmoor displayed evidence of the lowest genetic diversity.

Overall pony populations sampled from the U.S. showed lower levels of diversity than U.K. counterparts, particularly, the U.S. Fell and U.S. Dartmoor.

### Population gene flow and structure

**Figure 2:**
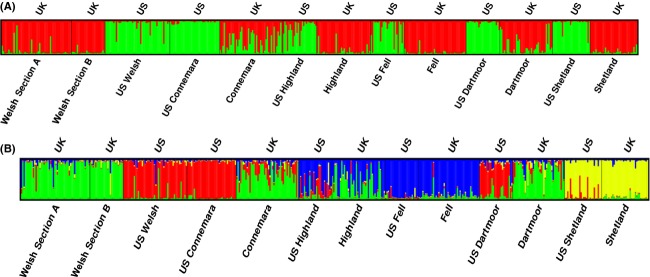
The results of STRUCTURE analysis for 10 SSR loci of each population for (A) *K* = 2 and (B) *K* = 4, averaged over six independent runs. STRUCTURE Harvester analysis suggests true value of *K* was 2 and 4.

Significant genetic structure was found within the dataset (*P* < 0.001), with 6.91% of total variation existing between populations within U.S. and U.K. groups. There was no significant variance found between U.S. and U.K. breeds. Nonetheless, Bayesian analysis using STRUCTURE of the log likelihood (Ln Pr(*X/K*)) of the posterior probability for a given *K* suggested the optimum value was *K* = 2. Delta *K* plots also showed a secondary peak for *K* = 4 clusters (Fig. 2A and B). At *K* = 2, clusters were divided according to geographic sample origin (U.S. and U.K.), with the exception of the Connemara. Where *K* = 4 clusters of sample origin were also evident, but the Fell and Shetland populations cluster together irrespective of geographical origin. Pairwise *F*_ST_ comparisons were performed based on the sum of squared allele size differences (*R*_ST_) and substantiated the relationship between populations of U.K. and U.S. origin (Table 2). Breeds did not show significant continental divergence except for Dartmoors, as is reflected in high estimates of Nm (migrants) between populations of the same breed. Of particular note was the Connemara with very low divergence between populations and a correlated Nm of 105.32. Similar to the summary statistics (Table 1), Theta *H* (4N_e_*μ*) values for autosomal data (Table 2) were reduced in most U.S. populations compared to U.K. counterparts indicating smaller effective population sizes, except for the U.S. Connemara and U.S. Welsh.

**Table 2:** Pairwise *F*_ST_ comparisons based on sum of squared size differences (*R*_ST_) and effective number of migrants for U.S. and U.K. pony populations

	Welsh Sect. A	Welsh Sect. B	U.S. Welsh	Fell	U.S. Fell	High.	U.S. High.	Conn.	U.S. Conn.	Dart.	U.S. Dart.	Shet.	U.S. Shet.
Welsh Sect. A	1.87	15.84	44.76	9.2	1.765	2.6	3.045	4.09	8.47	3.02	3.315	4.745	7.41
Welsh Sect. B	0.016	1.96	12.01	10.58	1.655	2.36	2.02	8.13	11.215	6.775	5.31	5.355	6.29
U.S. Welsh	0.006	0.02	2.25	0.037	1.505	0.099	3.05	0.069	0.038	0.101	3.15	0.077	4.8
Fell	0.026	0.023	6.535	2.08	13.26	5.755	13.9	7.68	11.82	3.83	4.29	3.29	3.34
U.S. Fell	0.124	0.131	0.142	0.019	1.81	0.081	3.67	0.089	0.081	0.139	1.13	0.153	1.195
High.	0.088	0.096	2.28	0.042	2.825	1.92	20.07	4.74	3.865	3.42	1.49	1.37	1.19
U.S. High.	0.076	0.11	0.076	0.018	0.064	0.012	1.72	0.069	0.056	0.119	0.17	0.157	1.46
Conn.	0.058	0.03	3.375	0.032	2.565	0.05	3.395	2.12	105.32	10.64	1.7	3.71	2.515
U.S. Conn.	0.029	0.022	6.27	0.021	2.855	0.061	4.255	0.002	2.18	0.028	2.46	0.044	4.725
Dart.	0.076	0.036	7.235	0.061	1.55	0.068	1.85	0.023	8.565	1.85	2.44	3.17	2.16
U.S. Dart.	0.07	0.045	0.074	0.055	0.181	0.144	1.225	0.128	0.092	0.093	1.67	0.152	1.725
Shet.	0.05	0.045	3.005	0.071	1.39	0.155	1.345	0.063	5.485	0.073	1.4	1.74	14.28
U.S. Shet.	0.033	0.038	0.05	0.07	0.173	0.173	0.146	0.09	0.05	0.104	0.127	0.017	1.68

Below diagonal: Pairwise *F*_ST_ based on sum of squared size differences (*R*_ST_). Above diagonal: effective number of migrants (Nm) per generation, based on Slatkin, 1995. Diagonal elements: Theta *H* estimates 4N_e_*μ* = (1/(1−*H*_e_)^2^)−1. Abbreviated populations are defined as follows: Welsh Section A, Welsh Sect. A; Welsh Section B, Welsh Sect. B; U.S. Welsh Pony, U.S. Welsh U.S.; Highland, High.; U.S. Highland, U.S. High.; Connemara, Conn.; U.S. Connemara, U.S. Conn.; Dartmoor, Dart.; U.S. Dartmoor, U.S. Dart.; Shetland, Shet.; U.S. Shetland, U.S. Shet. All values are significantly different (*P* < 0.05) with the exception of those in cells shaded grey.

Maternal-specific data are broadly consistent (Table 3), indicating more divergence among U.S. than U.K. populations (average Nm = 4.197 [*n* = 21] vs. Nm = 7.972 [*n* = 28]). Excepting the Fells and Dartmoors, transcontinental populations of the same breed did not show significant divergence in maternal genotypes as indicated by pairwise comparisons and estimated Nm (Table 3). However, the U.S. Welsh Pony, U.S. Connemara, and U.S. Highland exhibited particularly low divergence from other breeds of the U.S. and the U.K. (Table 3). Indeed, the U.S. Welsh Pony matrilineal lines show greater affinity with four U.K. breeds (Section A, Highland, Connemara and Dartmoor) than to the equivalent U.S. breeds, or indeed to that which the U.K. Section A shows to the other three U.K. breeds. U.S. Welsh Ponies showed the least distinction from, and highest number of migrants shared with, the Section B population. In contrast, the U.S. Fell pony was maternally significantly diverged from every other breed analyzed, including the U.K. Fell population. Possible admixture in some U.S. breeds, namely the U.S. Highland and U.S. Connemara, is indicated For example, the U.S. Connemara population showed twice as many migrants exchanged with the U.K. Section A than with the equivalent U.K. Connemara.

**Table 3:** MtDNA population average pairwise differences, within-population pairwise differences and estimated effective number of migrants

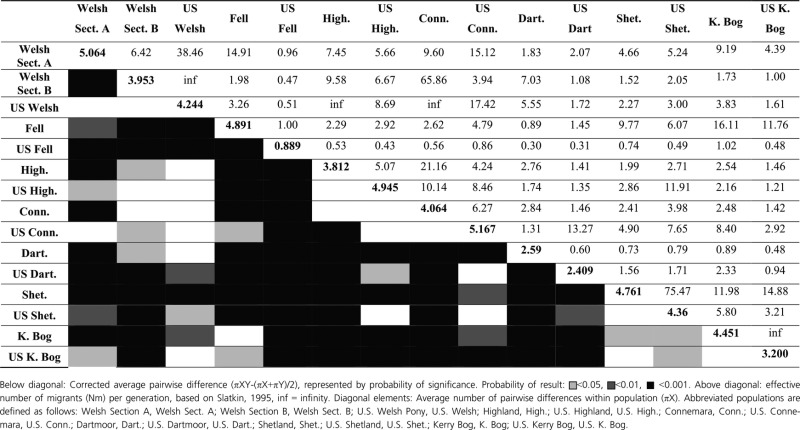

**Figure 3:**
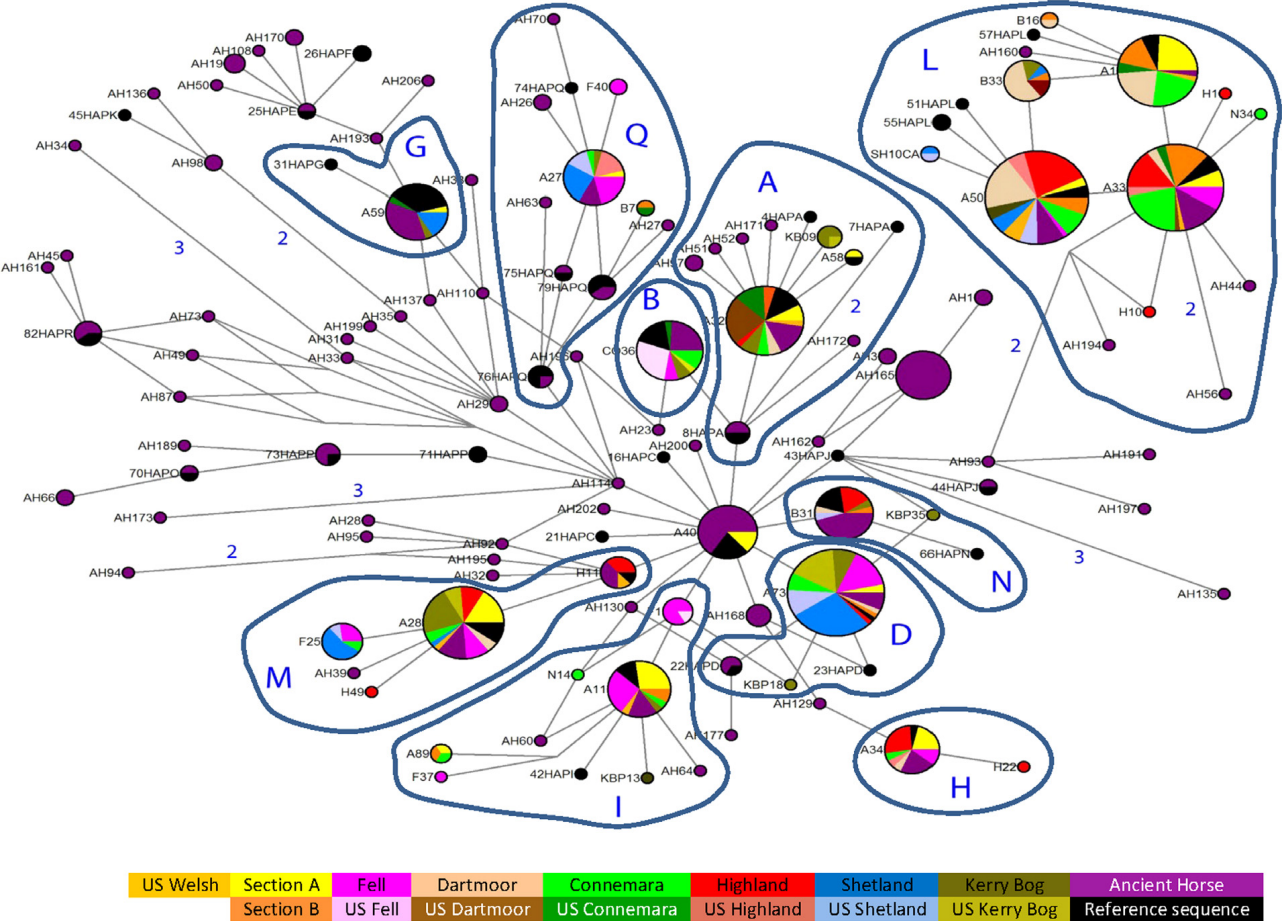
A median-joining network for mtDNA haplotypes displaying the relationships of the maternal lines of the British, Irish, and U.S. pony populations according to shared mitochondrial haplotype frequencies. Node size represents overall haplotype frequency with pie charts within nodes showing frequency of that haplotype by population. Reference samples representing examples of each of the major haplogroups identified by (Achilli et al. 2012) are displayed in black and labeled according to sample number and haplogroup, for example, “1HapA” Numerical values indicate the number of nucleotide changes (>1 mutation) between primary nodes. The haplotype designated as A40 is placed most ancestrally relative to the entire dataset and contains ancestral samples.

**Figure 4:**
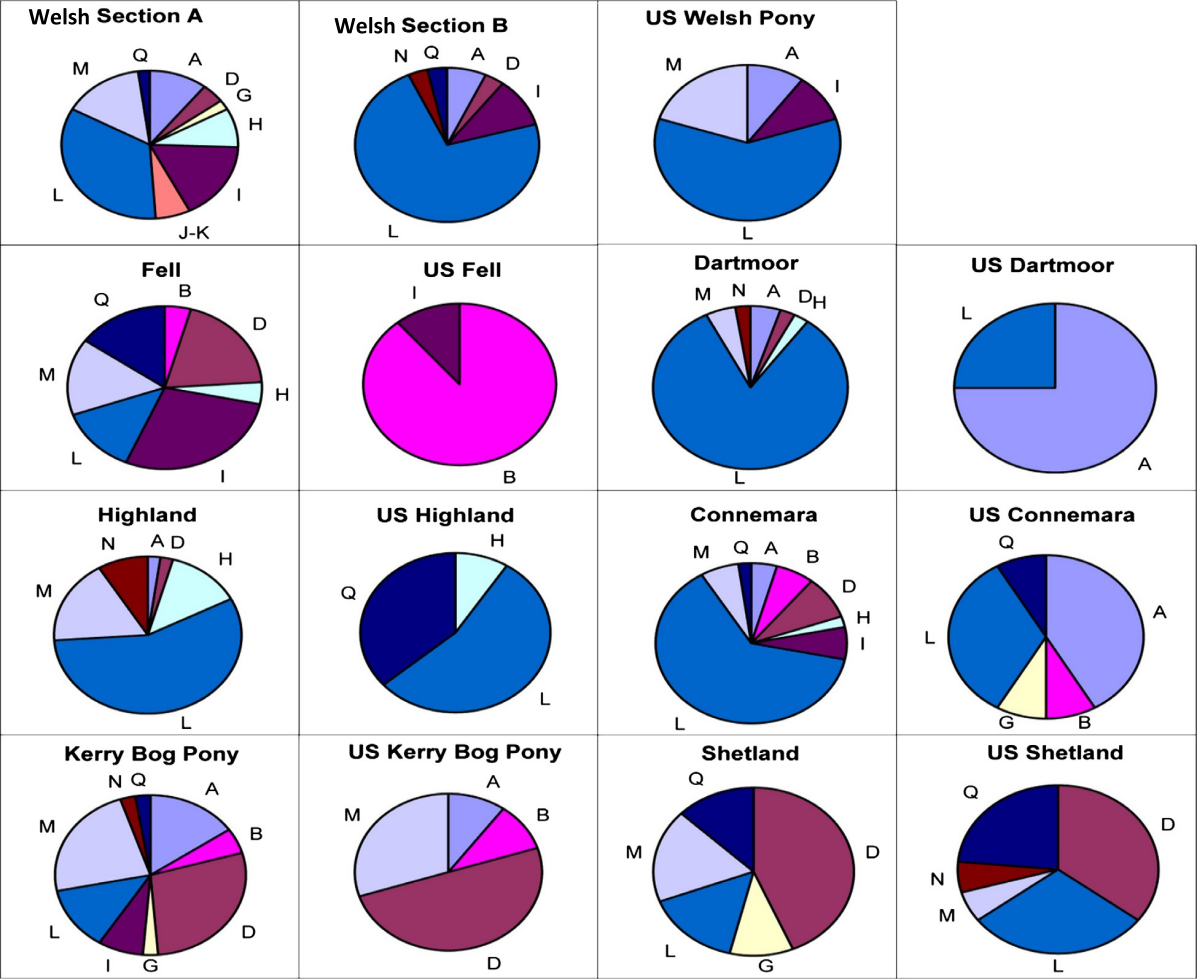
Haplogroup frequency (%) according to breed. Segments represent proportion of individuals belonging to labeled haplogroup. The first row identifiers “A” to “Q” represent the major worldwide mtDNA haplogroups in horses: haplogroup classification is based upon control region motifs, as described by (Achilli et al. 2012).

The median-joining network (Fig. 3) displays the relationships of the maternal lines of the British, Irish, and U.S. pony populations according to shared mitochondrial haplotype frequencies and using ancestral fossil samples to “root” the network. The U.S. Fell pony is noteworthy due to the overrepresentation of a single haplogroup: 89% of the U.S. Fells belong to haplogroup B, with only a single individual containing a haplotype found within another haplogroup (Figs. 3, 4). For some U.S. breeds, the haplogroup frequencies compared to the U.K. equivalent were similar (e.g., haplogroup L makes up 57% and 55% of the U.K. and U.S. Highland ponies respectively), but others show discrepancies (haplogroup A is present in 75% of the U.S., but only 5% of the U.K. Dartmoors, Fig. 3). In the more extreme cases, the “daughter” population contains haplogroups lacking from the U.K. source. For example, 36% of U.S. Highland samples contained the Q haplogroup that is absent from the U.K. Highlands, and similarly the haplogroup G sequences in U.S. Connemaras and haplogroup N in the U.S. Shetlands. Nonetheless, the general pattern is one of fewer haplogroups in the U.S. populations relative to the corresponding U.K. breed.

**Figure 5:**
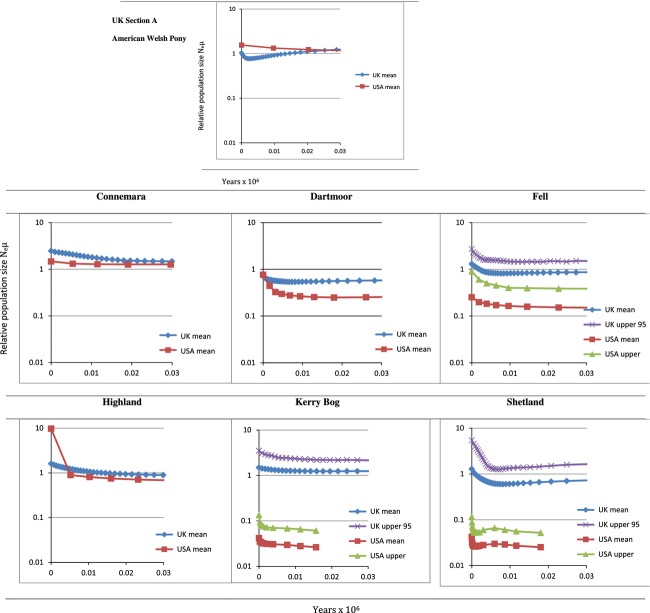
Extended Bayesian Skyline plots of estimated past population dynamics by Bayesian Monte Carlo simulation of coalescent molecular trees based on mitochondrial DNA data. The *y*-axis indicates the effective relative number of females, while the *x*-axis is years (×10^6^) before present. The plot lines are as follows: red, median estimate; blue, mean estimate; green, lower 95% limits; and purple, upper 95% limits. Note differing axis assigned to U.S. Welsh pony.

Skyline plots generated using mitochondrial sequence data illustrated that the effective population size of the U.S. breeds was lower than that of U.K. counterparts at a given time-point (*x*-axis) demonstrating the associated reduction in ancestral diversity as a result of a recent bottleneck in U.S. populations (Fig. 5 and Appendix 2). All populations appear to have undergone expansion, although for U.K. populations, this is estimated to have started substantially earlier than U.S. equivalents. Welsh populations did not show the same pattern as other groups, whereby the Section A showed a decline in effective population size and lower values than the U.S. Welsh population. Harpending's raggedness index test of the mitochondrial data for population expansion gave low raggedness index, indicating a unimodal distribution (Appendix 3). This generally supported the Skyline plots as unimodal distribution is found in populations having undergone recent population expansion. However, a significantly high (*P* < 0.05) raggedness index was found for the Welsh Section A, U.S. Connemara, Shetland, and U.S. Shetland population, which indicated that they existed in a multimodal distribution consistent with a stationary population for some time.

## Discussion

Establishing secondary ex situ populations to protect genetic resources is a common management tool in the conservation of a range of species (Fraser 2008; Ozer et al. 2011; Ransler et al. 2011; El Alqamy et al. 2012). For domestic livestock species, approximately 1080 breeds exist as “transboundary” populations, located internationally (DAD-IS, 2006). As an exemplar of whether a daughter population maintains the genetic diversity of its putative source once ex situ, our data suggest that pony populations sampled from the U.S. have lower levels of diversity than U.K. counterparts. However, specific exceptions exist for either mtDNA or SSR markers within certain breeds. The U.S. Fell and the U.S. Dartmoor have considerably reduced diversity for both mitochondrial and autosomal markers and seem to be descended from a low number of sires and very limited imported mares. Mitochondrial haplotype and nucleotide diversity within most of the other U.S. breeds was only slightly reduced compared to the U.K. populations. Based solely on these values, it may appear that most of the U.S. subpopulations have maintained much of the original maternal diversity of the U.K. populations they are derived from. However, closer examination of the mitochondrial haplotype distribution and population pairwise differences reveals discrepancies.

Certain haplogroups were present in the U.S. population, but absent in the U.K. equivalent for Highlands, Connemaras, and Shetlands, and although mtDNA and SSR diversities between U.S. breeds indicate more divergence than between the U.K. breeds, as expected for populations undergoing drift, there are some increases in lineage sharing between particular populations. In particular, the Welsh Section A, U.S. Connemara, U.S. Highland, and U.S. Welsh share more haplotypes than expected from other relationships possibly due to admixture. One interpretation of the intermediate probability of allocating Connemara animals to the U.S. or U.K. groupings in STRUCTURE plots is that this breed or the local derivatives have been crossed with other breeds in North America and to a lesser extent in the U.K. This may have occurred perhaps even before the respective stud books were closed (a closed stud book is one that prohibits registering animals whose parents were not themselves registered). Autosomal divergence between the U.K. and U.S. populations suggests that stochastic drift has occurred between source and daughter populations. The clustering according to breed rather than country of sample origin, evidenced by STRUCTURE analysis and pairwise *F*_ST_ comparisons (based on *R*_ST_ distance analysis), excludes this being the result of from laboratory-based genotyping discrepancies. Divergence between captive or wild subpopulations and source populations has been demonstrated for other species, where increasing gene flow or multiple restoration/reintroduction sources can limit the effects (Biebach and Keller 2009; Ozer et al. 2011; Wilson et al. 2012).

The Welsh breeds were an exception to the typical shared characteristics of an origin–daughter relationship observed within this study. It was not possible to identify an individual Welsh Section as the founder of the U.S. Welsh pony. The U.S. Welsh consisted of mitochondrial signatures that were shared by both U.K. sections As and Bs, yet the association with the Section Bs was most pronounced. This female amalgamation was also reflected in the Skyline plots whereby, unlike other U.S. populations, the U.S. Welsh pony showed an overall increase and a higher effective population size at the same relative time-point compared to the Section A. This is consistent with the U.S. Welsh pony sample used here having originated from a “mixed” population of Welsh sections A and B ancestors. As such, the U.S. Welsh population captures ancestral mitochondrial diversity from a larger population of origin than the U.K. Section A pony does alone. In effect, the bottleneck caused by breed formation was more recent in the Welsh Section As compared to the “mixed” origin of the U.S. Welsh. While cross-breeding and amalgamation seems to have occurred, particularly in the formation of the U.S. Welsh Pony, this contrasts with estimates of Nm for maternal data indicating American populations are more divergent from each other. This is therefore likely evidence of two processes. In one, founder and/or drift effects resulting in allele frequency differences (including loss of rare alleles) have driven populations in the U.S. apart and given lower estimates for previous population sizes due to loss of haplotypes passing through the bottleneck. In the other, cross-breeding or use of a common external breed (not included in the U.K. populations analyzed) as a crossing sire/dam in the U.S. has brought some of them together, but to a lesser degree than the stochastic changes that have caused the evident divergence.

As an example of this, SSR analyses demonstrated closer relationships between the U.S. Welsh and the Section A, with nearly four times the number of migrants exchanged than with the Section B, in contrast to the mtDNA patterns. It thus seems likely that U.S. Welsh ponies have a different maternal and paternal history; seemingly a product of mares from at least both sections of Welsh ponies from the U.K., while stallion introductions to the U.S. have been largely Section A. The diversity seen in the U.S. Welsh ponies today is therefore a consequence of original breed formation and subsequent repeated “migration” from one subpopulation of the source group. This highlights the importance for conservation strategies more generally to appreciate the degree of genetic variability and the level of gene flow among each subpopulation (Ciofi and Bruford 1999).

Skyline plots in the U.S. populations tend to be dominated by evidence of recent bottleneck effects, as only a limited signal of previous population changes can survive through the bottleneck to the current population. This is illustrated by mitochondrial haplogroup I, predominantly found in Section A and Fell ponies, but in only a single sample from each respective U.S. population. The branching star-shaped pattern of haplogroup I indicates these lineages may have existed within the earliest ancestral ponies of Britain and Ireland and is strongly characteristic of some of the more isolated populations (Winton et al. 2013). The “A40” node (Fig. 2) placed most ancestrally relative to the entire dataset is restricted to a substantial number of fossil samples and the Section A ponies. Similarly, the D haplogroup is rare worldwide (Achilli et al. 2012) and has been considered an old clade characteristic of the small ponies distributed on the western fringe of Europe (McGahern et al. 2006; Kakoi et al. 2007; Cieslak et al. 2010; Prystupa et al. 2012a) as confirmed in our U.K. populations. However, other than the recently imported U.S. Kerry Bog Pony and the U.S. Shetland, this rare haplogroup has been lost from the U.S. samples.

The recently characterized Kerry Bog is a prime example of the importance of consistency between conservation management organizations or breed societies. Our results indicate more restricted matrilines and lower diversity values in the U.S., but with no major divergence from the maternal source population. However, the recently established American Kerry Bog Pony Society accepts pied coloration not found in Ireland (Anon, 2013a); this divergence from the original breed indicating admixture is reflected in the lack of breed-specific clustering according to SSR analysis by Prystupa et al. (Prystupa et al. 2012b). While no SSR data currently exist for the U.K. Kerry Bog population, the introduction of pied-color animals will inherently change the genetic signature, causing greater divergence from the source population.

While statistically significant comparisons cannot be made for individual Skyline plots between the U.K. and U.S. populations, together they provide a comparative story of reduced population size and recent bottlenecks in the U.S. breeds. As expected, the results in Figure5 broadly indicate more recent establishment of U.S. populations and lower levels of subsequent importation resulting in larger discrepancies between predicted population sizes of the U.K. and U.S. breeds. The major exceptions are the U.S. Welsh and Shetland breeds. The U.S. Fell and U.S. Dartmoor pony populations consistently demonstrated major reductions in diversity compared to the original U.K. populations for both autosomal and mitochondrial analysis. Not only is there a marked decrease in relative diversity values, but also there is the significant overrepresentation of U.S. individuals within mitochondrial haplogroups that are rare within the respective U.K. populations, suggestive of severe population size restriction and consequent stochastic changes including the loss of ancestral matrilines (Lawler et al. 1995; Luis et al. 2006; Alvarez et al. 2012). Although this has resulted in significant mitochondrial divergence from the original U.K. populations, SSR analyses demonstrate a maintained link between autosomal allele frequencies among U.K. and U.S. Fells. Anecdotal records suggest that Dartmoor ponies, first introduced to the U.S.A. in the 1930s (Anon, 2013b,c), may have been maintained as a “pure” breed by mares derived from a single stud within the U.S., hence the divergence in autosomal and maternal markers. However, apparently extensive cross-breeding also occurred following U.S. import prior to the establishment of the Dartmoor Pony Registry of America in 1956; this may have occurred with local U.S. ponies, contributed further to allelic frequency divergence between the U.S. and U.K. groups (Anon, 2013b). The U.K. Dartmoor has also suffered reduced diversity as a result of historic demographic restrictions. As such, it is important that the ex situ population of this breed in particular is carefully managed to maintain maximal representation of the ancestral diversity, using methods such as avoidance of mate choice limitations and sex-biased skewing in reproductive success (Goyache et al. 2003; Eales et al. 2008; Conard et al. 2010; Brekke et al. 2011). The effects of various herd management strategies and the resulting variance in gene flow and admixture, applied here to different breeds, have resulted in a range of genetic signatures within the U.S. and U.K. subpopulations and indicate that recurrent importation has maintained genetic characteristics in some pony breeds, for example, the U.K./U.S. Connemara but has been less effective in others, such as the Fells.

## Conclusion

By studying ex situ subpopulations and putative source populations through the analysis of genetic diversity of ponies, we have established it is possible to identify different genetic characteristics that are compatible with known histories of those populations. We detected clear indications of founder effect and stochastic drift in most daughter populations, more pronounced in some breeds than others, and evidence of admixture of imported mares of different breeds within the U.S.A. To conserve a valuable resource and maintain sufficient adaptive genetic variability, it is important that derived populations remain representative of the original source once ex situ and that enough founder lines are present within the original or subsequently exported animals to maintain diversity levels and avoid extreme stochastic drift. It is imperative that the objectives of daughter breed societies/management groups are clear and consistent from the outset. We demonstrate that genetic analysis of subpopulations can assist in understanding the genetic effects of past management practices and inform future conservation strategies even in the absence of recorded histories.

## Appendix 1: Origin of data used within the study and GenBank sequences

**Table:**

	U.K. and Irish samples	North American samples	Kerry Bog samples	Ancient horse samples	Haplogroup identifier samples
SSR data	Original data generated for the current study and Winton et al. (2013)	Prystupa et al. (2012b)	NA	NA	NA
MtDNA data	Original data generated for the current, study, accession numbers KR361761–KR362244, and Winton et al. (2013)	Prystupa et al. (2012a); GenBank accession numbers HQ592784–HQ593063	McGahern et al. (2006); GenBank accession numbers DQ327852–DQ327890	Cieslak et al. (2010); GenBank accession numbers detailed in original manuscript “Supporting Information (Table S2)”	Achilli et al. (2012); GenBank accession numbers JN398377–JN398457

## Appendix 3: Harpending's raggedness index data (significant *P* values in bold)

**Table:**

Population	Harpending's raggedness index	*P* value (simulated raggedness ≥ observed raggedness)
Welsh Section A	0.0576	**0.01**
Welsh Section B	0.0423	0.64
U.S. Welsh Pony	0.0790	0.54
Fell	0.0179	0.42
U.S. Fell	0.7037	0.72
Highland	0.0547	0.13
U.S. Highland	0.1867	0.11
Connemara	0.0218	0.74
U.S. Connemara	0.1782	**0.05**
Dartmoor	0.0591	0.63
U.S. Dartmoor	0.4277	0.98
Shetland	0.1095	**0.01**
U.S. Shetland	0.3075	**0.02**
Kerry Bog Pony	0.0264	0.24
U.S. Kerry Bog Pony	0.1753	0.09

## Data Accessibility

Mitochondrial sequence data generated as part of this project are stored in FASTA format and will be uploaded to the NCBI/GenBank nucleotide sequence repository. Other mtDNA sequences incorporated into the analysis were downloaded from this source and can be retrieved as per the relevant citations. Breed-specific Nexus files were created for use in the Skyline analysis and will be made available via the DRYAD data repository. Processed SSR data will be also be uploaded to the DRYAD data repository as a tabular spreadsheet of allele scores with individual/breed/marker identifiers. This data was converted into several formats for use in packages such as STRUCTURE; should access to each individual format be deemed necessary, they will also be stored on the DRYAD repository. Raw SSR data (chromatogram peaks) are maintained in.fsa format and can be made available on request.
